# Role of Stimuli on Liquid Crystalline Defects: From Defect Engineering to Switchable Functional Materials

**DOI:** 10.3390/ma13235466

**Published:** 2020-11-30

**Authors:** Min Jeong Shin, Dong Ki Yoon

**Affiliations:** 1 Korea Advanced Institute of Science and Technology (KAIST), Graduate School of Nanoscience and Technology, Daejeon 34141, Korea; mjshin9@kaist.ac.kr; 2 Department of Chemistry, Korea Advanced Institute of Science and Technology (KAIST), Daejeon 34141, Korea; 3 KAIST Institute for Nanocentury, Korea Advanced Institute of Science and Technology (KAIST), Daejeon 34141, Korea

**Keywords:** liquid crystals, interface, surface treatment, rubbing, geometric confinement, electric field application

## Abstract

Achieving tunable physical properties is currently one of the most exciting research topics. In order to realize this goal, a medium that is responsive to external stimuli and can undergo a change in its physical property is required. Liquid crystal (LC) is a prominent candidate, as its physical and optical properties can be easily manipulated with various stimuli, such as surface anchoring, rubbing, geometric confinement, and external fields. Having broken away from the past devotion to obtaining a uniform domain of LCs, people are now putting significant efforts toward forming and manipulating ordered and oriented defect structures with a unique arrangement within. The complicated molecular order with tunability would benefit the interdisciplinary research fields of optics, physics, photonics, and materials science. In this review, the recent progress toward defect engineering in the nematic and smectic phases by controlling the surface environment and electric field and their combinational methods is introduced. We close the review with a discussion of the possible applications enabled using LC defect structures as switchable materials.

## 1. Introduction

Passing through the golden age of the liquid crystal display (LCD) industry, nanotechnology based on liquid crystals (LCs) started gradually fading away from the prime line in industries once organic light-emitting diodes (OLEDs) and quantum dot technology appeared in display applications. Albeit not the most exciting material anymore, LCs are still indispensable due to their intrinsic physical properties that are easily switchable under various external stimuli, including light [1,2,3,4,5,6,7,8], temperature [9,10,11], and electric [12,13,14,15,16,17,18] and magnetic sources [19,20,21]. As the current era demands novel materials with tunable physical properties in an on/off but dynamic manner, LCs can still be one of the most appropriate media to satisfy the demand.

Even though extant research using LCs is commonly based on the uniaxial alignment of molecules, it limits the variety that LCs can offer as a tunable medium. In addition to a uniaxially ordered and randomly oriented state, LCs can form defect structures with a distorted arrangement, which would find a meaningful role in vortex generation [15,22,23,24], diffraction grating [14,25,26,27], lithographic tools [28,29,30], and particle manipulation [15,16,28,31,32]. The defects can be considered useful only when formed on a large scale with order and controlled with stimuli such as temperature, light, or external fields. The requirement can be fulfilled by accompanying several techniques, including surface treatment, rubbing, geometric confinement, and external fields. The defect structures under control can be further manipulated with post-treatments using the techniques above to make larger contributions as a tunable medium.

In this review paper, studies on defect engineering in the last decade, exploiting surface treatment, rubbing, geometric confinement, electric fields, and combinational stimuli, are introduced. In the following section, useful applications based on the LC defect structures as a switching medium will be addressed. As the review focuses on the effect of the defect engineering techniques, research using the most conventional liquid crystalline phases, nematic (N), and smectic (SmA) phases, will be discussed.

## 2. Various Stimuli

**Figure 1:**
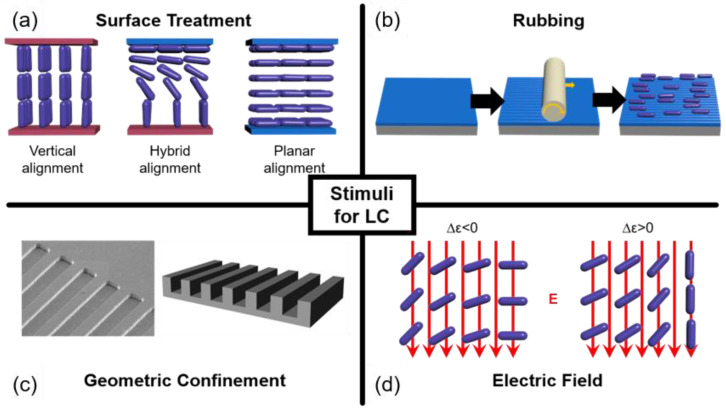
Types of stimuli used for defect engineering. (**a**) Surface treatment; (**b**) rubbing; (**c**) geometric confinement; and (**d**) electric field applications. The purple represents a liquid crystalline molecule. The red line in (**d**) indicates the direction of the electric field.

A typical LC molecule has a rod-like shape, consisting of a rigid core and flexible long tails. The structural anisotropy gives distinct dielectric and optical properties along a different axis, which allows the molecules to orient in response to the surrounding environment. Several techniques have been developed to control molecular orientation in the desired way. Stimuli acting under various circumstances are covered in this review. Surface treatment, rubbing, geometric confinement, and electric fields are the chief methods that determine the molecular orientation at the frontier while possessing universality in the LC fields (Figure 1). The effect of each stimulus is described below.

### 2.1. Surface Treatment

Changing surface polarity by treating a polymeric material on a substrate is the most convenient and mature way of controlling molecular arrangement. Polymers having either LC molecular-philic or -phobic properties are put on a substrate by the spin-coating method. Frequently encountered polymeric materials are polyimide (PI) [33], polyethyleneimine (PEI) [34], and polyvinyl alcohol (PVA) [35]. Some reported methods for surface treatment involve self-assembled monolayers (SAMs) [36,37].

**Figure 2:**
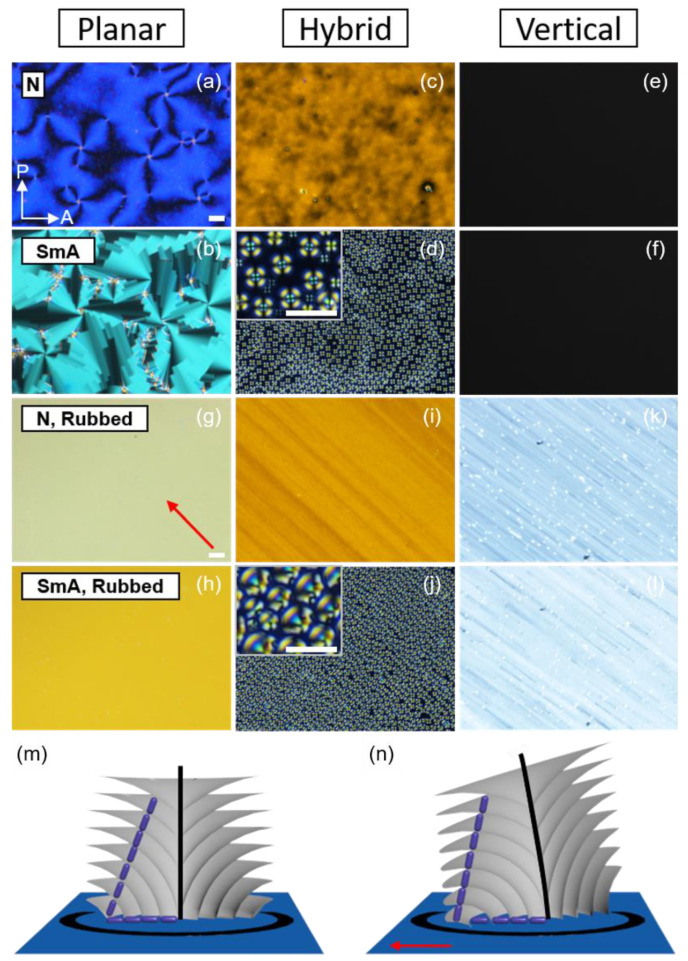
Polarized optical microscopic (POM) images under different boundary conditions in N and SmA phases with schematic illustrations of toric focal conic domains (TFCDs) and focal conic domains (FCDs). (**a**,**b**) Planar condition. (**c**,**d**) Hybrid condition. The inset of (**d**) is a magnified image. (**e**,**f**) Vertical condition. When rubbing is present, alignment along the rubbing direction (red arrow) is achieved. (**g**,**h**) Planar condition with rubbing. (**i**,**j**) Hybrid condition with rubbing where inset image shows a magnified view. (**k**,**l**) Vertical condition with rubbing. Schematic illustration of (**m**) TFCDs and (**n**) FCDs. The scale bar is 20 μm.

Three configurations can be obtained with the surface treatment: Vertical, hybrid, and planar alignments (Figure 1a). Vertical alignment refers to the arrangement of molecules whose long axis align perpendicular to a substrate, while planar alignment refers to the opposite. Hybrid alignment orients the long axis rotating by 90° from the top to the bottom substrate. Polarized optical microscopy (POM) images under different surface conditions are illustrated in Figure 2a–f. Depending on the surface polarity on the substrate, the LC molecules develop various optical features. Under the planar alignment condition, a typical schlieren texture is observed in the N phase, transforming into fan-shaped textures upon cooling to the SmA phase. The vertical alignment condition forces the molecules to stand with respect to the substrates, giving a dark optical texture. Especially in a hybrid condition, toric focal conic domains (TFCDs), typical smectic defects, are generated to satisfy the surface condition at each substate and the elastic deformation induced in the SmA phase. The Maltese cross pattern (inset of Figure 2d) implies that the layers are stacked parallel to the substrate, although the molecules are oriented radially at the bottom. TFCDs are a zero-eccentricity case of focal conic domains (FCDs) in which layers wrap around a conjugated ellipse and hyperbola line with eccentricity e, 0 ≤ e < 1 [28,38,39]. The schematic illustration of the molecular arrangement in TFCDs is addressed in Figure 2m.

### 2.2. Rubbing

Rubbing is a technique for achieving the uniaxial alignment of LC materials on the substrate [40,41,42,43]. A roller covered with a cloth revolves on a polymer-treated substrate to create tiny grooves and stretch the polymer chains of the alignment layer along the rolling direction (Figure 1b). Due to anisotropy on the substrate, the molecules tend to align along the rubbing direction. Figure 2g–l shows optical textures at three different surface conditions, and rubbing is applied on each substrate. Compared to the case where rubbing is absent, uniform domains without any defect structure are obtained except for the hybrid anchoring condition. For the molecules situated in the hybrid anchoring condition, the uniaxial grooves give rise to the development of tilted FCDs that align their ellipse and hyperbola along the rubbing direction in the SmA phase (Figure 2n). The rubbing method is especially useful for materials with the N phase in their phase sequence, as this phase possesses a more fluidic nature than any other LC phase. However, smectic phases with a 2-dimensional order can still be affected by rubbing due to the strong influence of the molecular organization in the N phase toward the organization in the SmA phase. Ok et al. [44] reported the multi-rubbing induced spatial arrangement of TFCDs, which was enabled by a tendency to maintain organization in the N phase once transitioned to the SmA phase. Sharing the function of guiding molecular orientation with the rubbing method, a photo-alignment technique has become popular [45,46,47,48,49]. It can overcome the drawbacks of the rubbing, including the mechanical damage and electrostatic charges. With this method, various azimuthal organizations of the molecules can be achieved, so that complicated or hierarchical defect structures can be easily generated. Research using the photo-alignment technique will not be covered in this review, as many studies are still rooted in the traditional rubbing method owing to its universality.

### 2.3. Geometric Confinement

Since Choi et al. [50] reported the exploitation of microchannels to generate periodic smectic defects, many experimental results using microchannels have been obtained [17,28,33,34,51,52,53,54,55]. As LC materials should adopt a specifically defined orientation under limited volume and surface conditions of microchannels, the so-called confinement effect, they develop a peculiar structure in a periodic manner. The increased surface area to volume ratio under the geometric confinement also contributes to LC molecules’ being strongly influenced by surface boundary conditions. Therefore, microchannels and other geometry-inducing confinement effects are employed to investigate the nature of defect structures in various LC phases, including N, SmA, cholesteric, and even Bn phases [33,35,56,57,58,59,60,61,62,63,64,65].

**Figure 3:**
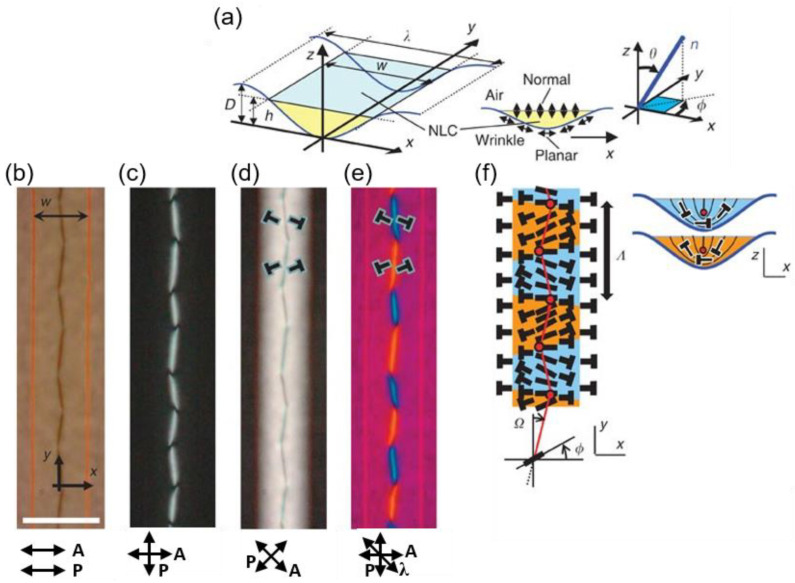
Periodic zigzag line defects under sinusoidal micro-wrinkle confinement. (**a**) Illustration of the micro-wrinkle system and its surface boundary condition. D is the depth of the micro-wrinkle, h is the thickness of the Liquid crystals (LCs), λ is the wavelength of the micro-wrinkle, and w is the width of the LCs. The blue line on the right indicates the orientation of the LCs, where θ and φ are polar and azimuthal angles of the local director n, respectively. (**b**–**d**) POM images of periodic zigzag lines are observed under various configurations of the polarizers (P and A). In (**e**), a λ plate is inserted at 45° with respect to the crossed polarizers to provide detailed information of the molecular organization. The blue line indicates the local molecular director is parallel to the λ, and the yellow line indicates that it is perpendicular. The symbol T shows the projection of the director onto the plane of the page, where its end is going inward through the page. The scale bar is 20 μm. (**f**) Schematic illustration of molecular arrangement in the micro-wrinkle confinement. φ is a twist angle of the director n with respect to x, while Ω is a twist angle of the disclination line from the y-axis. Λ denotes the periodicity of the periodic structures. The red dots correspond to the disclination kinks. Reprinted with permission from [32], Copyright 2012, Springer Nature.

Generation of periodic zigzag lines in the N phase is possible when a nematic material is subjected to sinusoidal confinement with antagonistic surface anchoring [32]. The sinusoidal micro-wrinkle grooves cause the molecules to be preferentially aligned in the x-z plane and along the groove’s tangential direction to satisfy homeotropic anchoring LC-air and planar alignment at the LC-groove interface with the easy axis along the x-axis. Under this condition, the LC material shows periodic twist deformation and zigzag line defects (Figure 3b–f). This is the result of competition between intrinsic anisotropy in the elasticity of the LC material (K_2_ > K_1_ > K_3_, where K_1_, K_2_, and K_3_ are elastic constants for splay, twist, and bend, respectively [66]) and boundary conditions of the groove. The smaller twist elastic constant (K_2_) in the N phase drives the formation of twist deformation below the disclination line, finally leading the line defect placed away from the groove center. These molecular orientations give rise to the observed periodic zigzag line (Figure 3f).

**Figure 4:**
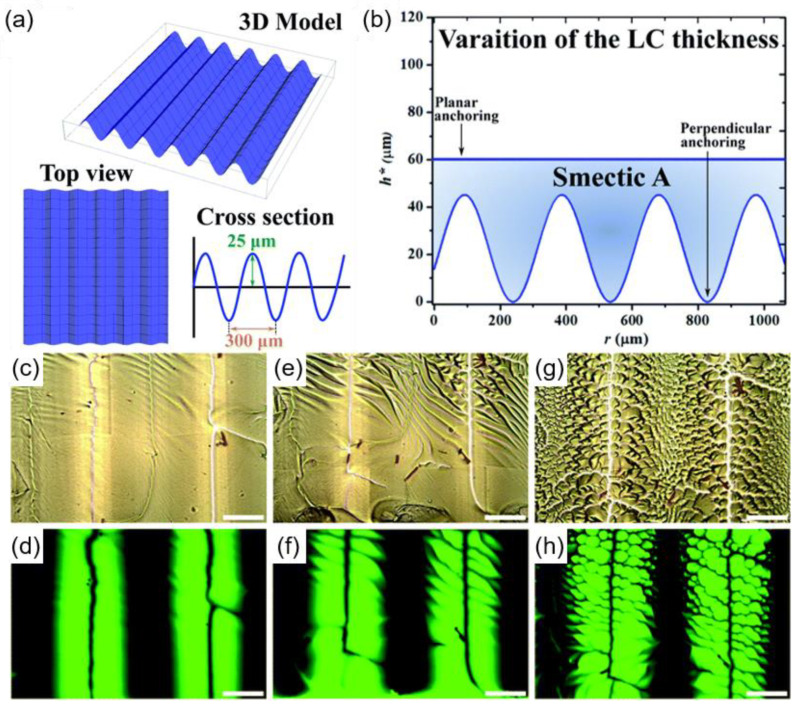
Smectic FCDs organized under sinusoidal micro-wrinkle confinement. (**a**) 3D illustration of the micro-wrinkle system with a period of about 300 μm and an amplitude of about 25 μm. (**b**) The surface anchoring condition at undulating interfaces and a graph showing the variation of the LC thickness. (**c**–**h**) Formation of FCDs across the N-SmA phase transition. (**c**–**d**) Bright field and fluorescence image of disclination line in the N phase. (**e**–**f**) Bright field and fluorescence image in the N-SmA phase transition. (**g**–**h**) Bright field and fluorescence image of FCDs in the SmA phase. The scale bar is 100 μm. Reprinted with permission from [35], Copyright 2020, Royal Society of Chemistry.

**Figure 5:**
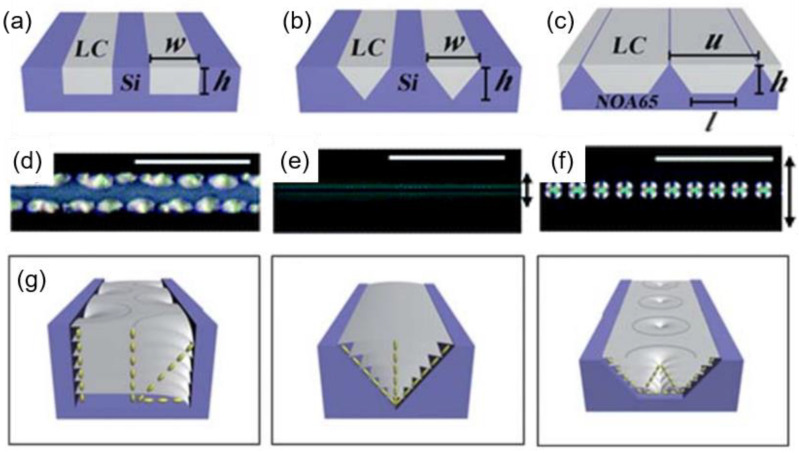
Smectic defects under various geometric confinements. The confinement systems, respectively, have (**a**) a rectangular shape, (**b**) a V-shape, and (**c**) a trapezoidal shape. POM images obtained in (**d**) rectangular, (**e**) V-shaped, and (**f**) trapezoidal channels. The double-headed arrow denotes the width of the channel. Schematic illustration for layer orientation and molecules are addressed in the case of (**g**) rectangular, (**h**) V-shaped, and (**c**) trapezoidal channels. The scale bar is 50 μm. Reprinted with permission from [53], Copyright 2011, Royal Society of Chemistry.

Another example of using sinusoidal micro-wrinkle confinement presents the fabrication of hierarchical assembly of FCDs in the SmA phase [35]. In this case, the surface boundary conditions are the opposite of the those found in the research mentioned above (Figure 4a). An LC material exhibiting N and SmA phases is confined between a base of cross-linked polydimethylsiloxane (PDMS) and a coverslip. The PDMS, having topographic undulations, has a homeotropic anchoring condition, while the coverslip is treated with PVA, which induces planar anchoring. The cell geometry causes thickness variation in the LC materials, as indicated in Figure 4b. In the N phase, straight disclination lines positioned at the crests and troughs of the underlying geometry are observed. Cooling to the SmA phase stabilizes the disclination lines, with the formation of FCDs facing opposite directions between the lines (Figure 4c–h). However, flat substrates with the same anchoring condition could not position the disclination lines in a periodic manner or stabilize them, as it disappears spontaneously to reduce the total free energy. Introduction of confining topographic pattern and fine control on the anchoring condition will allow disclinations to be located and will create a new defect arrangement. Interestingly, the topographic pattern also contributes to altering the size and eccentricity of FCDs, which results in a hierarchical arrangement of defects. Conclusively, the size and eccentricity of FCDs are highly related to the thickness variation of the LC materials. Larger FCDs with higher eccentricity are observed in thicker regions and vice versa. This shows the importance of confining geometry on the formation and modulation of the defect structures. The effect of geometry on LC defect formation is shown in Figure 5. Kim et al. reported a periodic arrangement of TFCDs along the middle of trapezoidal microchannels, while the other geometry could not accomplish the same ones [53]. Due to the antagonistic boundary condition, semi-circular TFCDs are generated in a rectangular channel. In the case of the V-shaped channel, the pointy bottom cannot afford space for the molecules to be arranged radially, so it is not possible to generate defect structures. However, a trapezoidal channel could produce TFCD arrays along the middle area. The molecules are preferentially aligned tangentially to the bottom and inclined toward the sidewall, satisfying the surface anchoring condition, with enough volume to create the defects.

**Figure 6:**
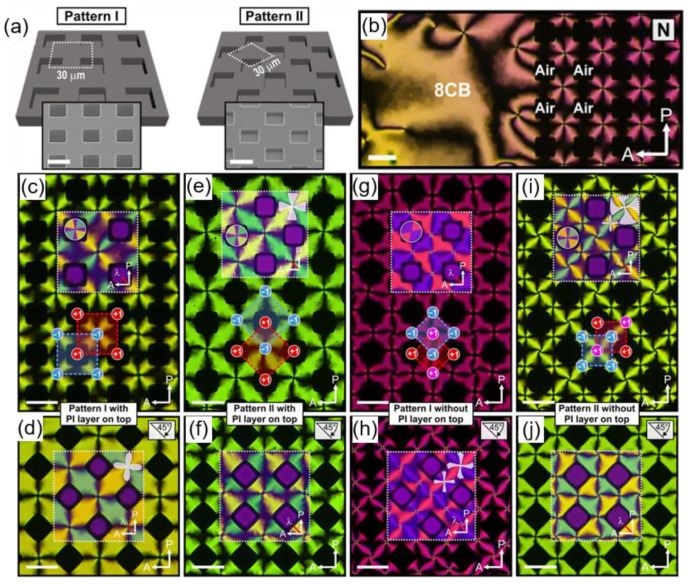
Checkerboard patterns of alternating +1 and −1 defects achieved by using air pillar arrays as underlying substrates. (**a**) Pattern I and Pattern II, having simple square tiling and truncated square tiling, respectively. (**b**) POM image of 8CB in a planar surface boundary condition (left) and on the topographic pattern (right). (**c**–**j**) POM images of the periodic checkerboard pattern in the N phase obtained with or without the PI treatment on the cover glass. −1 defects positioned at the center of each square in Pattern I with a PI layer on top (**c**–**d**) and Pattern II with a PI layer on top (**e**–**f**). +1 defects in the middle of each square and −1 defects between each pair of pillars are created in Pattern I without a PI layer on top (**g**–**h**) and Pattern II without a PI layer on top (**i**–**j**). The inset images correspond to POM images obtained by inserting a λ plate. POM images of the same structures as above are taken under 45° rotated crossed polarizers and given in the bottom row (**d**,**f**,**h**,**j**). The scale bar is 20 μm. Reprinted with permission from [63]. The Authors, some rights reserved; exclusive licensee American Association for the Advancement of Science. Distributed under a Creative Commons Attribution Noncommercial License 4.0 (CC BY-NC) http://creativecommons.org/licenses/by-nc/4.0/.

Figure 6 depicts periodic defect arrays produced by using air pillars as a confining system [63]. Air pillars created between a hollow relief and flat substrate can act as confining interfaces with a strong homeotropic anchoring condition. This condition imposes topological frustration on LC molecules, which then produce two-dimensional periodic textures. The N phase placed in the air pillar system shows a checkerboard pattern of alternating +1 and −1 defects (Figure 6b). The strong homeotropic anchoring from the air pillar induces radial alignment around them with winding number +1, which stabilizes neighboring −1 defects at the same time. To examine the effect of the geometry, two kinds of patterns are used, denoted as Pattern I and Pattern II. The lattices of Pattern I and Pattern II are repeated at a different angle, 45°. In both cases, checkerboard patterns with −1 defects at the center of each square of four neighboring air pockets are obtained. The geometry, in this case, has little impact on modulating defect structures. Additionally, two kinds of surface treatments on the coverslips are used: One coated with PI for inducing the strong degenerate condition and the other without any alignment layer. In this trial, the density of defects could be varied. The strong anchoring of the PI-treated substrate leads to a decrease in the number of defects because of the high free energy penalty of the defects, while the untreated one is rather free from this constraint.

**Figure 7:**
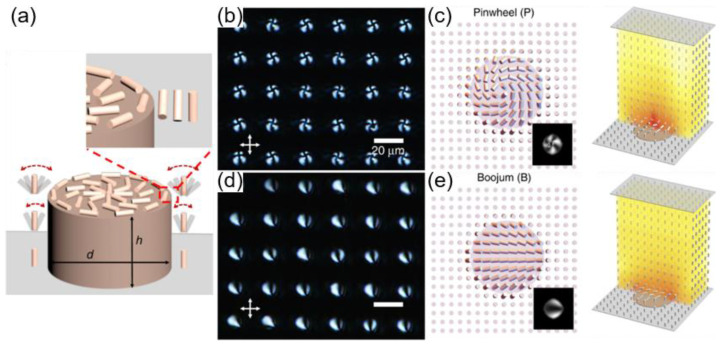
Periodic pinwheel (P) and Boojum (B) defects on circular posts. (**a**) Schematic illustration of liquid crystals on a circular post. Three-dimensional distortion is induced at the post’s edge. (**b**) POM images of pinwheel textures. (**c**) Director configuration of the pinwheel at the top of the post (left) with simulated POM images (inset) and three-dimensional reconstruction (right). (**d**) POM images of Boojum textures. (**e**) Director configuration of Boojum at the top of the post (left) with simulated POM images (inset) and three-dimensional reconstruction (right). The scale bar is 20 μm. Reprinted with permission from [67], Copyright 2019, Springer Nature.

The last example of using geometric confinement on defect engineering is illustrated in Figure 7 [67]. Compared to the previous cases, the geometry provokes three-dimensional distortion of the molecular directors, particularly at the post’s edge. The experimental system comprises a substrate with a square array of circular posts (d = 10 μm, h = 0.2 μm) and a cover slide (Figure 7a). While the underlying and top substrates induce a homeotropic anchoring condition, the circular posts impose a degenerate planar anchoring condition. Here, another type of deformation, referred to as saddle-splay deformation, should be considered. Saddle-splay deformation is known to occur when molecular directors are varied in at least two orthogonal dimensions. In the N phase, two kinds of defect structures are generated when heating and cooling are repeated (Figure 7b–e). The first is a periodic pinwheel configuration, where +1 defects are positioned at each post with breaking achiral symmetry (Figure 7b–c). The second corresponds to the boojum, where a defect resides at the edge of the post (Figure 7d–e). One of the two structures mentioned above is achieved with high spatial uniformity at each heating and cooling sequence. However, either of the two structures is generated randomly, indicating that they are at about a similar energy level with kinetic differences in each cycle leading one or the other. Uniform configuration with the diagonal alignment of directors can also be achieved when the post’s height is increased to 2 μm. Therefore, specially designed topographic patterns with additional chemical treatment could generate three-dimensional molecular distortion that results in various defect structures stabilized with high uniformity.

### 2.4. Electric Field Application

Rod-shaped LC materials with distributed charges, in general, are responsive to the applied electric field. The difference in polarizability, thus in permittivity (Δε), along the long and short molecular axis determines how the LC would behave under an electric field. If the LC material has positive dielectric anisotropy, the molecules tend to align their long axis parallel to the field direction. Materials with negative dielectric anisotropy would align perpendicular to the field. This feature of LC material led to flourishing in the LC research field, paving the way toward developing the LC display (LCD). The stimuli introduced in previous sections can only achieve static profiles of defect structures, whereas using the electric field enables dynamic modulation on defect structures by changing voltage or frequency.

**Figure 8:**
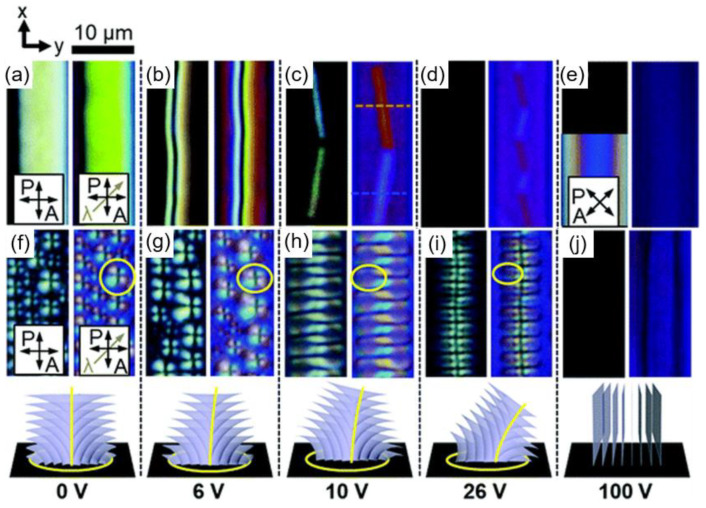
Structural transformation of nematic and smectic defects under in-plane electric field application. (**a**–**e**) POM textures in the N phase when the electric field is increased from V = 0 V to V = 100 V. The inset of (**e**) is obtained under 45° rotated crossed polarizers. (**f**–**j**) POM textures in the SmA phase when the electric field is increased from V = 0 V to V = 100 V. The bottom row corresponds to the internal structure of the smectic defects at each electric field condition. The yellow circle in (**f**) represents the focal curves of the TFCD, and yellow circles in (**g**–**i**) indicate elliptic-hyperbolic focal curves of the FCD. The gray arrow represents a λ plate. The scale bar is 10 μm. Reprinted with permission from [16], Copyright 2019, Royal Society of Chemistry.

Suh et al. recently reported the dynamic modulation of disclination lines and FCDs in the N and SmA phases, respectively, by changing the magnitude of voltage (Figure 8) [16]. The LC material having both N and SmA phases with positive dielectric anisotropy is confined under a hybrid anchoring condition with an applied electric field in the in-plane direction (y-axis). The arising aligning force of the electric field would gradually orient the molecular long axis toward the y-axis. In the N phase, a wedge disclination line pinned at either edge of electrodes is transformed first into the zigzag structure and then to the uniaxial alignment upon the increase in voltage up to V = 100 V (Figure 8a–e). As twist deformation is preferred over other deformations in the N phase, the zigzag structure is generated at a moderate voltage condition. Further increase in voltage results in a uniform domain where all molecules are aligned parallel to the field direction. In the case of the SmA phase, a layered defect structure is dynamically modulated (Figure 8f–j). TFCDs formed under the initial hybrid condition are changed to alternating FCDs, face-to-face FCDs, and uniaxial orientation, respectively, upon increasing electric fields. Due to the lowest K_1_ (splay constant) value in this phase, FCDs embedding splay deformation are likely to be formed. The external field then causes the molecules to incline toward the bottom substrate along the y-axis with increasing voltage, thereby suppressing splay deformation. The defect generation under this condition could be controlled by changing the voltage and the temperature.

**Figure 9:**
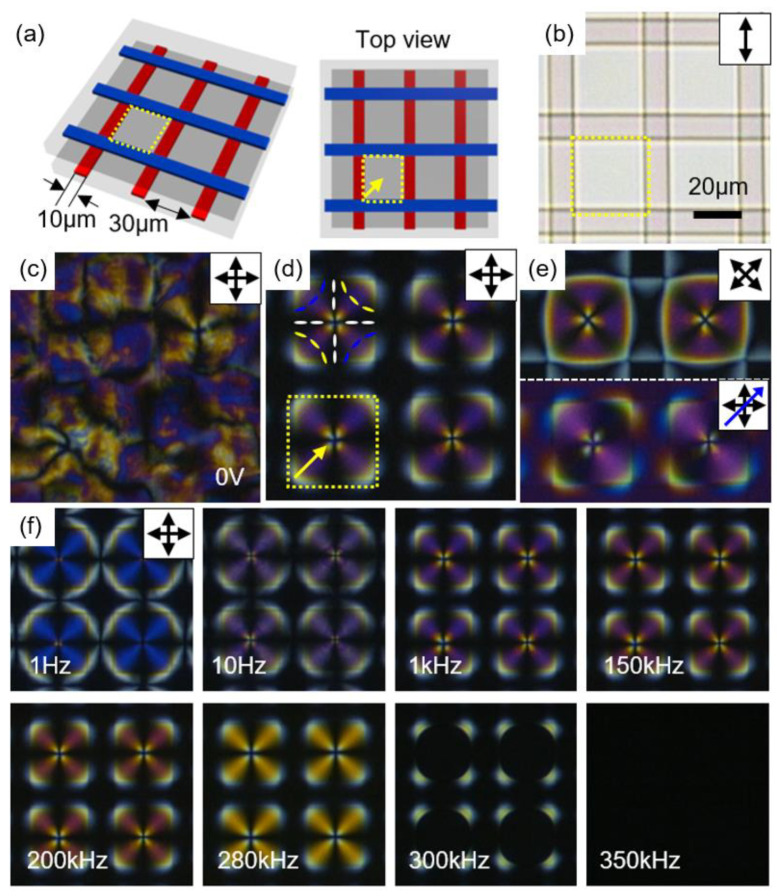
Periodic LC defect arrays generated by the crossed electrodes. (**a**) Schematic illustration of the cell geometry, where red and blue lines indicate top and bottom electrodes, respectively. (**b**) OM image of the crossed electrode cell. (**c**) POM image obtained without electric field application. (**d**) Periodic and uniform LC defect structures under an applied electric field (V = 30 V, f = 3 kHz) in the N phase. (**e**) The same structure observed under 45° rotated crossed polarizers and with λ plate (blue arrow) insertion (below). (**f**) Structural change driven by altering frequency at a fixed voltage (V = 30 V). The yellow dotted line identifies a unit cell where the −1 defect resides at the center. The unit cell refers to the area surrounded by the crossed electrode strips. The yellow arrow shows the growth direction of the textures (from the electrode edge to the center). Reprinted with permission from [15], Copyright 2019, Wiley-VCH.

A unique geometric design for electric field applications can induce the generation of periodic defect arrays over a large area [15]. Figure 9 describes how exotic defect structures having four-fold symmetry with a point defect at the center are created and manipulated with the electric field. The cell geometry is achieved by combining two in-plane switching (IPS) cells at the right angle (Figure 9a,b). As the patterned indium-tin-oxide (ITO) substrates are untreated, they possess weak degenerate planar anchoring condition, giving a schlieren texture in the N phase (Figure 9c). When sufficient voltage V_0_ = 30 V at f = 3 kHz is applied, periodic defect arrays including −1 topological defects are created (Figure 9d,e). Accordingly, +1 topological defects are positioned above the crossed electrodes, conserving the total topological charge to zero. Furthermore, the retardation color of this pattern could be dynamically controlled by varying frequencies. For example, as can be seen in Figure 9f, the retardation color is changed from indigo to violet, purple, deep-red, orange, yellow, and white in order. The trend in color change coincides with the reduced retardation values in the first-order region in the Michel-Levy birefringent chart [68]. This modulation in retardation color, Γ, originates from dielectric heating [69,70]. At high frequency, a dielectric relaxation process induces an increase in temperature within the sample, resulting in decreased effective birefringence. The same trend in retardation color upon increasing temperature showed the same result.

**Figure 10:**
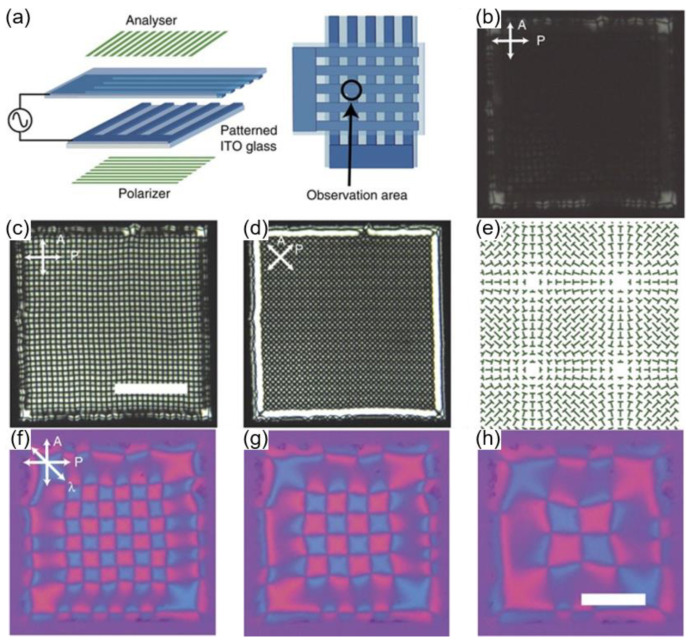
Large-scale self-organization of LC defects. (**a**) Schematic illustration of the cell geometry comprising top and bottom electrode at 90°. (**b**) POM image of the initial stage of the formation of grid-texture. (**c**) POM image of the grid texture and (**d**) the same texture observed under 45° rotated crossed polarizers. The scale bar is 200 μm. (**e**) Schematic illustration of the director profile. (**f**–**h**) Tunable defect spacing by volage modulation. POM image with a λ plate inserted when (**f**) V = 17.5 V, (**g**) V = 26.2 V, and (**h**) V = 39.3 V. The scale bar is 100 μm. Reprinted with permission from [71], Copyright 2016, Springer Nature.

As shown in Figure 10, a similar cell geometry design is employed to generate highly ordered defect arrays [71]. In this case, a nematic material, having negative dielectric anisotropy with ion impurity, is used. The molecules are aligned perpendicular to the electric field. This means a degeneracy exists on the azimuthal angle, thereby creating topological defects on the plane parallel to the substrates. The experiment is performed by applying an AC voltage to the square regions formed on crossed electrode stripes (Figure 10a). Starting from the homeotropic alignment at a low frequency, periodic umbilic domains are generated along the edge of the square with increasing frequency (Figure 10b). Epitaxial growth is then initiated from the umbilics at the edge, finally resulting in a single domain of the umbilic defects (Figure 10c–e). Dynamic manipulation on the defects is also possible by alternating the electric field. It is shown that defect spacing increases with varying voltage, which implies that the number of defects in a given area can also be modulated (Figure 10f–h). Therefore, electric field application is an effective stimulus on LC defect structures because it allows for the generation of exotic structures and dynamically manipulates them. 

### 2.5. Combinational Methods

In this section, recent research using combinational methods is addressed. As described above, each stimulus serves its unique function in the molecular arrangement. Unfortunately, it has been considered difficult to use multiple triggers together due to physical limitations; for example, rubbing cannot be applied to the geometric confinement system. Therefore, additional efforts involving lithographic techniques should be employed.

**Figure 11:**
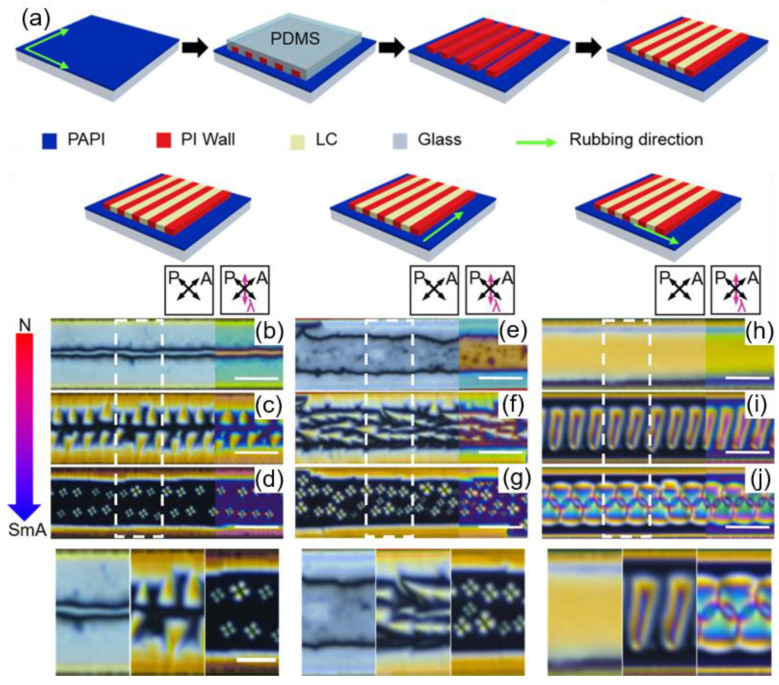
Directed assembly of topological defects using a combinational method of surface anchoring and geometric confinement. (**a**) Experimental scheme generating an antagonistic boundary system with confinement and rubbing. POM images of smectic defects on cooling, confined in an antagonistic boundary microchannel, where rubbing is (**b**–**d**) absent, (**e**–**g**) parallel with the microchannel, and (**h**–**j**) perpendicular to the microchannel. Right figures of each image show POM obtained with a λ plate inserted. The scale bar is 20 μm. The bottom figures correspond to magnified images of the white-dashed box; the scale bar is 10 μm. Reprinted with permission from [33], Copyright 2018, American Chemical Society.

Via a soft-imprinting method, an LC alignment platform, imposing the simultaneous effects of surface anchoring, rubbing, and confinement, can be produced, as Shin et al. reported (Figure 11) [33]. A confining geometry is introduced with an imprinting method in which a polymeric mold is used. Two additional variables, the anchoring condition and the rubbed surface anchoring condition, are controlled. Figure 11a depicts the experimental scheme, in which there are antagonistic surface anchoring conditions at the sidewalls and the bottom. Different rubbing conditions on the bottom substrate can also be established: Non-rubbed (Figure 11b–d), rubbed in a parallel direction (Figure 11e–g), and rubbed in a perpendicular direction (Figure 11h–j) with respect to the microchannel. On a substrate with planar anchoring, PI-based microchannels, exhibiting vertical anchoring, are imprinted. These polymer-based channels act as surface anchoring agents and provide confinement at the same time. Due to the homeotropic anchoring condition in the air and at the sidewalls, parabolic FCDs are generated along the sidewalls [51,52,72]. Depending on the rubbing condition, the morphologies of the smectic defects are controlled, switching from TFCDs, to tilted TFCDs, to zigzag structures, and packed with periodicity. Hence, the ensemble of multiple stimuli grants the ability to control defect morphology in a versatile way.

**Figure 12:**
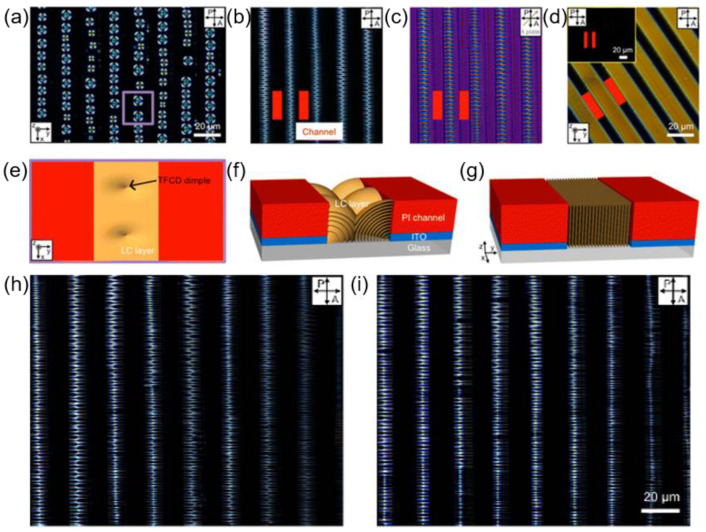
LC structures confined in the polymer microchannel where the electric field condition is varied. (**a**) TFCDs of the SmA phase placed along the middle of the channel. (**b**) Zigzag patterns under the electric field (V = 30 V and f = 1 kHz). (**c**) POM image of the zigzag patterns after insertion of a λ plate (gray arrow). (**d**) Linearly oriented layers along the electric field direction (V = 100 V and f = 1 kHz); inset is the POM image when the channel direction is parallel to the polarizer. Schematic diagrams of (**e**) TFCDs, (**f**) the zigzag structures, and (**g**) the linearly oriented layers, respectively. (**h**) The zigzag pattern under the electric field (V = 30 V, f = 1 kHz) and (**i**) after removing the electric field. Reprinted with permission from [17], Copyright 2016, American Chemical Society.

Beyond the surface anchoring and confined geometries, electric field-driven dynamic change in the smectic defect structure has been accomplished via a combination of confinement and electric field application (Figure 12). In this work, line-type PI microchannels are placed on IPS mode electrodes. At the initial state, regularly packed TFCDs are initially formed along the channel due to the combinational effect of confinement and surface anchoring anisotropy between the air and the channel walls (Figure 12a,e). Upon application of the in-plane electric field, morphology change takes place on defect structures. When a mild electric field (V = 30 V and f = 1 kHz) is applied, the flexoelectric effect of the smectic director resulting from a fringe field of the electrodes mainly determines molecular arrangement (Figure 12b,c,f) [73,74]. Intrinsic to the SmA phase, the splay flexoelectric effect in response to the electric field results in zigzag structures. The confinement role here is packing the defects with the periodicity and stabilizing frustrated structures even after removing the electric field (Figure 12h,i). Under the strong electric field (V = 100 V and f = 1 kHz), however, the field-induced dielectric polarization of LC materials is dominant, and the molecules are uniaxially aligned along the field direction (Figure 12d,g). The combined effect of confinement and the electric field could control layering topographic patterns by changing the field strength.

**Figure 13:**
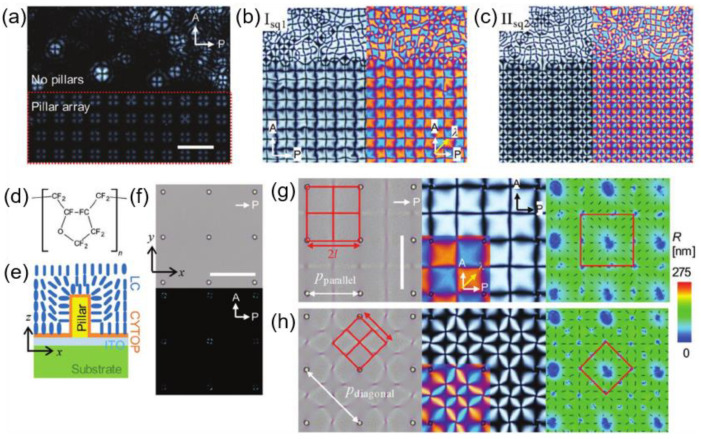
Dynamic topological defect pattern formation using micropillar arrays and electric field application POM images observed at (**a**) V = 14.4 V and f = 220 Hz, (**b**) V = 15 V and f = 370 Hz on pattern I_sq_, and (**c**) V= 25 V and f = 190 Hz on pattern II_sq_. The arrow, denoted as λ, indicates an inserted direction of a λ plate. (**d**) The chemical structure of CYTOP, which imposes homeotropic anchoring on LC. (**e**) Schematic of LC director fields around a pillar. (**f**) OM (top) and POM (bottom) images with no electric field. (**g**–**h**) OM (left), POM (middle), and PolScope (right) images showing light retardation integrated across the cell thickness with molecular distribution (black bars). (**g**) At V = 45 V and f = 120 Hz. (**h**) At V = 22 V and f = 300 Hz. The red square defines a primitive unit cell of each case. The scale bars are 100 μm in (**a**–**c**) and 50 μm in (**f**–**h**). Reprinted with permission from [14], Copyright 2020, Wiley-VCH.

Along with the microchannels, pillar arrays have also revealed their tremendous defect manipulation capacity under certain conditions [14]. Figure 13 describes a pillar-assisted generation of periodic defect structures that are tunable with the electric field. The surface anchoring condition is designed as homeotropic so that the pillar serves as a strong attractor for +1 radial topological defects (Figure 13e). By applying the electric field, umbilical defects are formed, arranging +1 and −1 defects alternatively between the pillars (Figure 13a–c). Due to the pre-determined nucleation sites on the pillars, the LC defects are organized with periodicity over a large area. Moreover, the spacing between defect points and the repeating direction of primitive cells, defined by the smallest repeating unit, can be modulated by changing the frequency and the applied electric field simultaneously (Figure 13g–h). This research proves the importance of the combinational contribution of the surface treatment, pillars, and electric field because it would not be possible to obtain the tunable periodic defect arrays without any one of them.

## 3. Applications

In this section, possible applications using defect structures are introduced. LC materials accommodate their intrinsic elasticity and surrounding environment to produce specific patterns whose regularity or shape can be adjusted with various methods, as mentioned above. The fabricated highly ordered LC defect patterns can find their value in some research areas, including particle manipulation and electro-optic devices.

### 3.1. Particle Manipulation

**Figure 14:**
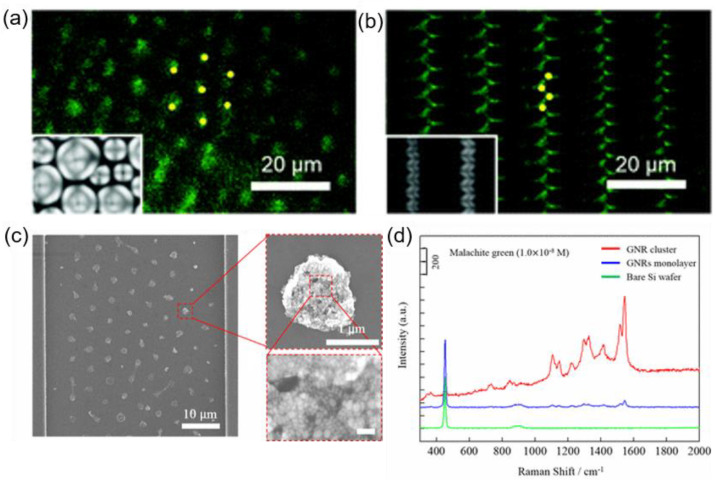
Defect-driven particle manipulation and SERS. (**a**,**b**) Fluorescence microscopy images of quantum dot (QD) clusters collected at defect sites. (**a**) QD clusters positioned at the center of the TFCD structures at V = 0 V. (**b**) Zigzag arrayed QD clusters at V = 14 V. Reprinted with permission from [16], Copyright 2019, Royal Society of Chemistry. (**c**) SEM images of gold nanorods (GNRs) trapped in TFCDs after removing sublimable LCs with magnified images of clusters (red dashed boxes on right) and (**d**) SERS spectra of Malachite green (MG) on a GNR cluster array (red), a GNR monolayer (blue), and a bare Si wafer (green). Reprinted with permission from [79], Copyright 2018, American Chemical Society.

The LC defect structures have been exploited as a template for trapping colloidal particles such as nanoparticles and quantum dots (QDs) in previous studies [75,76,77,78]. Trapping phenomena occurs because placing particles at LC defects reduces the overall free energy of the system. Significantly, the TFCDs possess a dimple of about 100 nm due to layer bending toward the bottom at the center, which implies that it can provide a reservoir for particles. As indicated in Figure 8, smectic defect structures can be dynamically manipulated. When QDs are introduced into the system, tunable QD arrays can be obtained by modulating electric field strength. In the absence of the electric field, TFCDs are formed, thereby collecting QDs at their centers (Figure 14a). Upon turning on the electric field (V = 14 V), alternatively packed FCDs are created, and QDs are trapped at the domain boundary of the FCDs whose directors are mostly disorganized (Figure 14b).

Periodic TFCD arrays are also generated using a sublimable LC material in microchannels (Figure 14c) [79]. When gold nanorods (GNRs) are incorporated in the LC phase, they preferentially place themselves at the center of TFCDs to form clusters. The LC’s subliming nature allows for the removal of LC material by thermal treatment, leaving clusters of GNRs at the position that have been occupied by TFCDs previously. The leftover GNR cluster arrays can be used for SERS applications (Figure 14d). The analyte used here is Malachite green (MG), a banned fish pesticide. Significant amplification of the peak intensity of the Raman peak is detected for the substrate with the arranged GNRs, compared to the signal noticed on the flat gold substrate or with the monolayer of GNRs. This implies that the LC defect-driven clustering of GNRs has led to an enhancement of Raman signals, compared to the unpatterned GNRs.

### 3.2. Switchable Electro-Optic Devices

**Figure 15:**
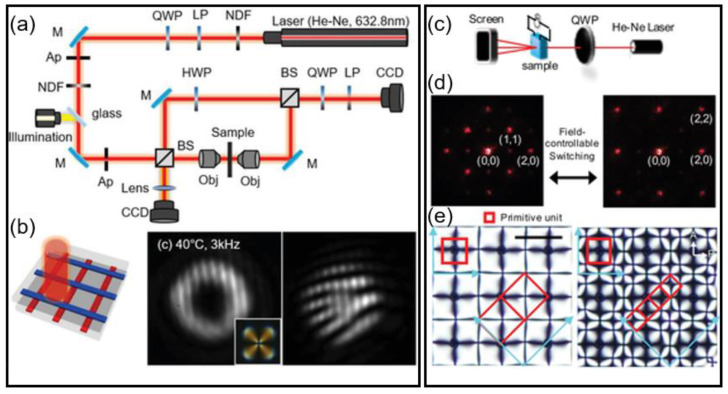
Application toward tunable electro-optic devices. (**a**,**b**) Reconfigurable LC defects for vortex beam generation. (**a**) Schematic illustration of experimental setup. The abbreviations are as follows: NDF, neutral density filter; Ap, aperture; BS, beam splitter; M, mirror; LP, linear polarizer; QWP, quarter-wave plate; Obj, objective lens; CCD, charge-coupled device camera. (**b**) Beam projection through the unit cell (left), the resultant beam profile (middle), and interference pattern (right). The inset image is a corresponding POM image of the −1 defect. Reprinted with permission from [15], Copyright 2019, Wiley-VCH. (**c**–**e**) Dynamically controlled LC defects for the tunable diffraction grating. (**c**) Optical setup. (**d**) Field-controllable 2D diffraction patterns obtained at V = 50 V, f = 120 Hz (left), and V = 25 V, f = 280 Hz (right). (**e**) Their corresponding POM images, with red boxes indicating primitive units. Index of diffraction spots are provided in (**d**), where (0,0) implies a spot of the central laser beam. Reprinted with permission from [14], Copyright 2020, Wiley-VCH.

Regularly arranged defect arrays also play a significant role in developing switchable electro-optic devices. Here, applications toward a vortex beam generator and a diffraction grating are addressed. The defect arrays consisting of periodic −1 defects, illustrated in Figure 9, show an ability to generate a vortex beam when illuminated with circularly polarized light (CPL) (Figure 15a,b) [80,81,82]. It has been recognized that LC cells with a spatial modulation of directors can generate a vortex beam because it can act as a geometric phase. For the incident CPL, it experiences a handedness change, accompanied by a phase shift of 2θ, where θ is an angle between the fast axis of the wave plate and the plane of polarization after passing through the geometric phase. Here, the −1 defects with fourfold symmetry around a point defect transform the incident beam into the vortex. The interference pattern with a reference beam is investigated to analyze the phase distribution of the transmitted vortex beam (Figure 15a). In this research, the pattern achieved at conditions where V = 30 V, f = 3 kHz, and temperature = 40 °C gives rise to a typical vortex beam profile with the highest conversion efficiency (Figure 15b). The fork-shaped interference pattern with two dislocations confirms that the transmitted vortex carries a phase topological charge of +2 (−2) for the incident right CPL (or left CPL) [83,84]. This proves the topological charge of the LC defect has −1. The conversion to the vortex beam is switched off by either increasing frequency or temperature due to randomly arranged molecular alignment in the Iso phase.

The periodic defects that are tunable with the electric field, as shown in Figure 13, can be used as a switchable 2D diffraction grating (Figure 15c–e). The diffraction patterns under the electric field-driven modulation in defect arrays are interchangeable. Upon changing the field condition, the defect spacing is altered. It induces a change in the diffraction angle (α) because the spacing (Λ) is inversely proportional to α, as Λ~ λ/(2sinα) [85]. Moreover, the repeating direction of the primitive cell can be changed from parallel to diagonal, which induces in-plane rotation of the diffraction patterns. The switching between the defects and the diffraction patterns is reversible and repeatable due to the electric field-responsive property of the LC material.

### 3.3. Microlens Array

**Figure 16:**
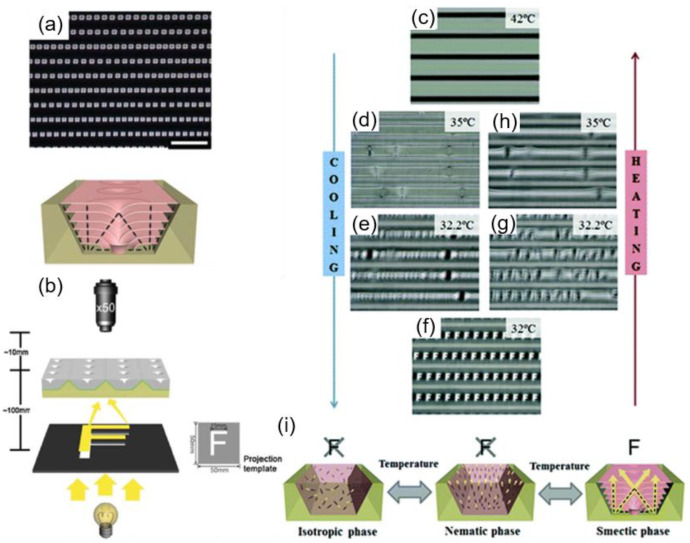
Application toward a thermally reversible microlens array. (**a**) POM image of periodic TFCD arrays along the trapezoidal microchannels (top) and the illustration of the cross-sectional internal structure (bottom). (**b**) Experimental setup for fabricating microlens arrays. The focal plane of the microlens arrays are determined by scanning the sample in the z-direction. A black polymer sheet with a transparent letter “F” is placed below the sample. (**c**–**f**) OM images taken during cooling and heating sequences, showing the thermally reversible ON/OFF behavior of the microlens arrays. The cooling and heating rate is maintained at 1 °C min^−1^. The letter “F” is clearly observed in the SmA phase (**f**). (**i**) Schematic illustration on how the microlens arrays are responsive under the thermal change. Reprinted with permission from [86], Copyright 2012, Royal Society of Chemistry.

Confining smectic LCs in a trapezoidal channel results in a single array of TFCDs along the channel (Figure 16a) [53,86]. The intrinsic molecular arrangement, composing a TFCD, allows incoming light to be focused at the center due to the gradually-increased local effective refractive index in this region. The microlens effect of the periodic TFCD arrays has been analyzed (Figure 16b) [86]. A transparent letter “F” (size: 15 mm × 15 mm) on a black sheet is placed below the defect arrays. As the channel is made of a transparent polymer, the incident light from the template can be projected through the TFCDs. When the focal point of the objective lens matches the focal plane of the TFCDs, the letter on the template can be clearly observed. The phase transition to the N and Iso phases enables the microlens to be switched off while the system can recover its functionality once the smectic defects are formed (Figure 16c–i). No diffracted image is observed due to the absence of focusing units in the N and Iso phases. Consequently, the periodic TFCD array, which is reconfigurable under temperature control, allows a thermally responsive microlens array to be fabricated with a simple confinement method.

## 4. Conclusions

In this review, a recent approach to obtaining controlled defect arrays as well as the applications of these defects are introduced. In the first part, we discuss techniques of manipulating molecular orientations at the interface. Surface treatment and rubbing methods were used in the early stage of LCD development, but variation in those techniques has opened a new field of LC research. This variation involves additional stimuli such as geometric confinement and electric fields, which create a more frustrating environment on LC molecules. As a result, a wide variety of defect structures can be created and manipulated by controlling parameters, such as the geometric shape and the electric field condition. The applications of these defects are described in the next part. Defects created under control find a role in particle manipulation, electro-optic devices, and microlens arrays. The intrinsic stimulus-responsive characteristics of LC allow for tunability in those applications. The system can be manipulated when the embedded defects are changed, for example, when vortex beam generation is switched on/off by increasing frequency. Therefore, research on defect engineering emphasizes the importance of choosing the right stimuli for generating the desired pattern and thus for controlling it on demand. Although this review covers the most accessible techniques for defect engineering, there is still more to be discussed due to the diversity in liquid crystalline phases and stimuli. Therefore, researchers must devise novel techniques for controlling molecular structures and thus expand their use in many other kinds of research, including optics, photonics, and energy [87,88,89,90,91,92].
